# Harnessing the Heterogeneity of T Cell Differentiation Fate to Fine-Tune Generation of Effector and Memory T Cells

**DOI:** 10.3389/fimmu.2014.00057

**Published:** 2014-02-19

**Authors:** Chang Gong, Jennifer J. Linderman, Denise Kirschner

**Affiliations:** ^1^Department of Computational Medicine and Bioinformatics, University of Michigan, Ann Arbor, MI, USA; ^2^Department of Chemical Engineering, University of Michigan, Ann Arbor, MI, USA; ^3^Department of Microbiology and Immunology, University of Michigan Medical School, Ann Arbor, MI, USA

**Keywords:** two-compartment model, lymph nodes, blood, agent-based, circulation, systems biology, dendritic cells, cognate

## Abstract

Recent studies show that naïve T cells bearing identical T cell receptors experience heterogeneous differentiation and clonal expansion processes. The factors controlling this outcome are not well characterized, and their contributions to immune cell dynamics are similarly poorly understood. In this study, we develop a computational model to elaborate mechanisms occurring within and between two important physiological compartments, lymph nodes and blood, to determine how immune cell dynamics are controlled. Our multi-organ (multi-compartment) model integrates cellular and tissue level events and allows us to examine the heterogeneous differentiation of individual precursor cognate naïve T cells to generate both effector and memory T lymphocytes. Using this model, we simulate a hypothetical immune response and reproduce both primary and recall responses to infection. Increased numbers of antigen-bearing dendritic cells (DCs) are predicted to raise production of both effector and memory T cells, and distinct “sweet spots” of peptide-MHC levels on those DCs exist that favor CD4+ or CD8+ T cell differentiation toward either effector or memory cell phenotypes. This has important implications for vaccine development and immunotherapy.

## Introduction

**Figure 1:**
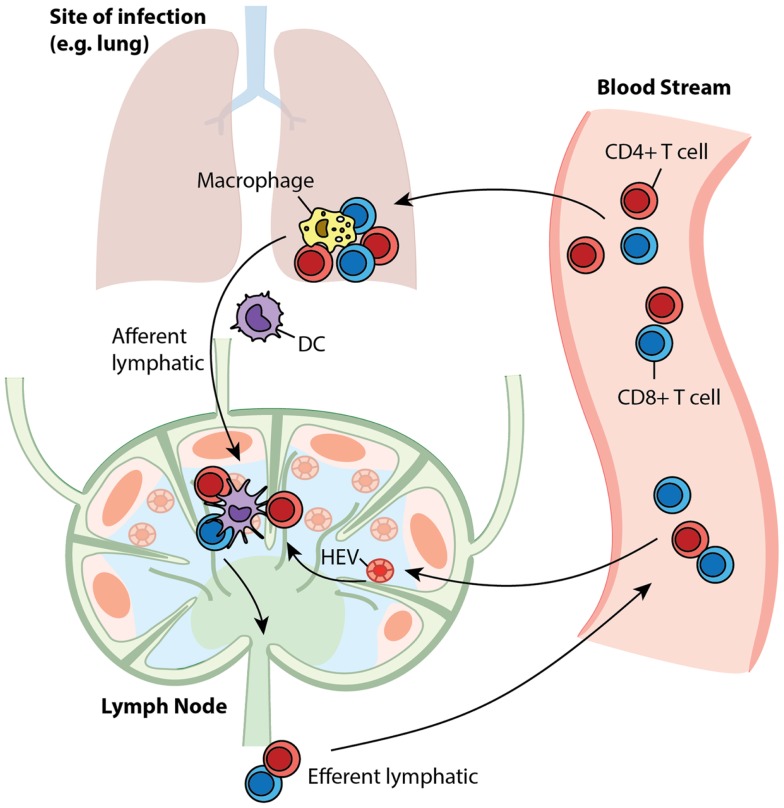
**T cell trafficking between compartments**. Naïve T cells circulate between LNs and blood. Upon infection, APCs present antigen to cognate T cells in LNs to initiate their proliferation and differentiation to generate effector and memory cells. After entering the blood, effector cells are recruited to sites to fight ongoing infection, while memory cells recirculate, awaiting secondary infections.

Antigen-presenting cells (APCs), especially dendritic cells (DCs), process antigens and carry information from sites of infection to secondary lymphoid organs, such as lymph nodes (LNs) (1). T cells are produced in the thymus and are deployed into blood circulation to recognize millions of different epitopes from pathogenic organisms; each T cell is hardwired to have one type of T cell receptor (TCR) that recognizes a single pattern (i.e., “cognate” with respect to a specific antigen) (2). The frequency of particular cognate T cells is as low as 10^−5^–10^−6^ (3, 4). Through high endothelial venules (HEVs), T cells are recruited to LNs, where they are exposed to antigenic peptides presented by MHC molecules expressed on DCs – this initiates the adaptive immune response (5–9). LNs are organized such that when T cells travel through they can be efficiently scanned by DCs to identify that rare cognate encounter (10–12). Such encounters result in binding of cognate T cells to DCs and subsequent activation and proliferation of the T cells. The expanded T cell population differentiates into two classes: effector cells, which perform immediate killing and cytokine secretion functions, and memory cells, which are reserved for long-term protection (13, 14). These cells move out of LNs via efferent lymphatics (ELs) into blood circulation (15). Through the blood, effector T cells reach sites of infection while memory T cells continue to recirculate and await a potential secondary infection for which they will wage a faster and stronger recall response (16, 17). A snapshot of the trafficking of these cells is shown in Figure 1. The immune system responds differently to different antigenic materials; however, the same set of machinery is engaged to face each challenge. Thus, there should be a general program adaptively guiding the behavior of this system. In this study, we focus on cellular-mediated events shared among immune responses during the initiation of adaptive immunity and generation of immune memory.

Differentiation of T cells during generation of adaptive and memory responses is highly heterogeneous, and this heterogeneity is may be dependent on the environmental context that each cell experiences (18, 19). However, the cause of such heterogeneity is poorly understood. If mechanisms other than mere stochasticity contribute to heterogeneity, it could be possible to more precisely direct the differentiation to favor the production of the desired output from an immune response (e.g., effectors in an immune therapy or memory cells in vaccination) by manipulating the mechanisms involved. We are interested in which mechanisms could provide handles for such manipulation. Since T cell priming occurs in LNs, and blood circulation conveys effector and memory T cells to locations where they perform their specific functions, mechanisms in these two organs could be responsible for the heterogeneous differentiation. The dynamics of T cells in these compartments will also reflect progression of infection or effectiveness of vaccinations. Thus, understanding how different LN and blood mechanisms affect the dynamics of infection and treatment could help guide immunotherapy and vaccine design.

Computational and mathematical models are widely used in biological systems to assess hypotheses and generate predictions for experimental validation. Deterministic equation-based models have been developed to understand the dynamics of T cells responding to immunogenic antigens, and these models helped with estimating parameters, determining alternative hypothesis, and predicting the outcomes of immune responses (20, 21). Agent-based models (ABMs) have proven convenient in assessing roles of cellular and molecular level interactions during infection (22–27). However, because of the extremely low cognate frequency that exists in primates, these models usually require large numbers of cells to be simulated and thus are very computationally intensive. In order to capture both heterogeneous stimuli-sensitive short-term activation events as well as average long-term dynamics, a model needs to be capable of adapting itself to both situations.

In this study, we present a hybrid computational model that uses an agent-based modeling to capture events occurring in a LN and a non-linear ordinary differential equation (ODE) model to capture events occurring in the well-mixed compartment of blood. This model allows us to track a highly stochastic immune response operating during the first few weeks of an immune response (with time resolution around seconds), as well as long-term dynamics afterward (at a time scale of months to years). Using this model, we assess which mechanisms in both LN and blood compartments control the differentiation and clonal expansion processes of T cells and also direct the immune response toward potent effector T cell output and/or robust memory generation. These findings could bring insights to vaccine design strategies.

## Materials and Methods

### LN ABM model

Agent-based models are computational models in which individual agents are represented on an explicitly formulated grid and they interact with each other according to a defined set of rules implemented in discrete time steps. As these types of models can account for spatial-sensitive interactions between DC and cognate T cells, they are ideal for studying heterogeneous priming and differentiation of T cells in LNs (23–25, 28, 29).

We previously developed *LymphSim*, a three-dimensional (3D) LN computational model capturing dynamics of CD4+ T cells, CD8+ T cells, and DCs during both steady state and infection (30). Briefly, cells move on a 3D grid that is shaped like a truncated cone and represents ~1/200 of a primate LN. T cells enter the LN via HEVs, search for DCs, activate and proliferate to generate effector cells that exit via ELs. In *LymphSim*, cell motility and steady state values in a LN are calibrated to experimental data with model antigens such as OVA (31), and the dynamics during an immune response are not quantitatively fit to any specific infection. For simplicity, we only include one type of cognate T cell each for CD4+ and CD8+ T cells in current model, and DCs present the corresponding antigens on pMHC (peptide-MHC)-II and pMHC-I for both primary and secondary infections. The model can be adapted to account for multiple sub-antigens. For the work herein, this single antigen study is sufficient to address the key questions under study. A complete list of rules can be found at: http://malthus.micro.med.umich.edu/lab/movies/3dLN/.

### Effector and memory T cell differentiation rules

**Figure 2:**
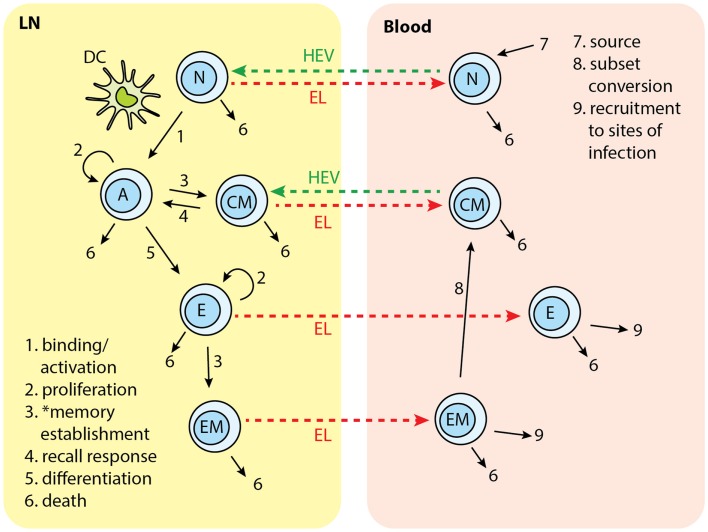
**T cell subsets in two-compartments of LNs and blood: N, naïve; A, activated; CM, central memory; E, effector; EM, effector memory**. Each number indicates a collection of processes occurring in that step and in different cell types. Naïve T cells are recruited to LN from blood. In the LN, cognate T cells bind with Ag-DCs and get activated. Activated T cells proliferate and differentiate into central memory (CM) and effector cells. CM in the LN can bind to DC and be activated again. Effector T cells can further differentiate to effector memory (EM) cells. Naïve, effector, CM, and EM exit LN from EL. Naïve and CM cells recirculate between LN and blood. Effector and EM are recruited to sites of infection. EM can covert to CMs. *Memory establishment for CD8+ T cells requires LDCs.

In the present study, we modified *LymphSim* to include two additional T cell differentiation states: central memory (CM) and effector memory (EM), for both CD4+ and CD8+ T cells. We also added rules that govern generation of these memory cells, and their interaction with other cells (Figure 2).

**Figure 3:**
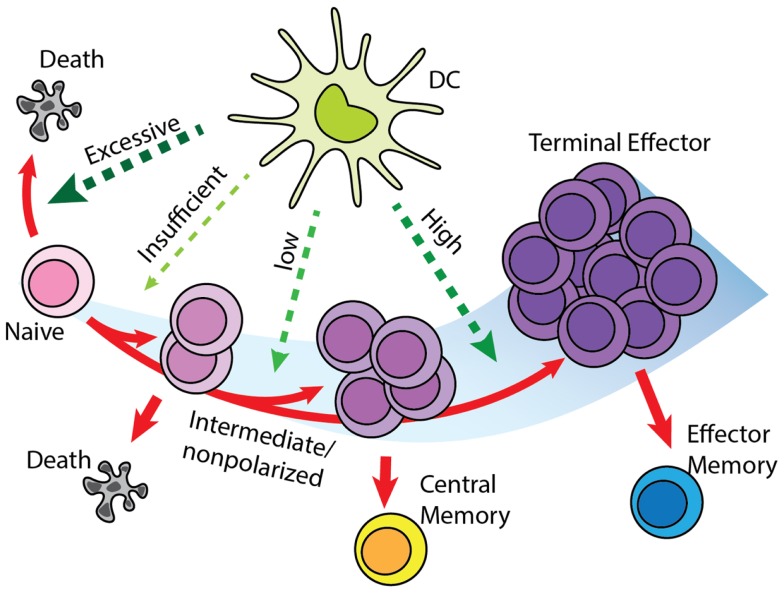
**“Signal-strength model” of T cell differentiation**. T cells receive antigenic, co-stimulatory, and inflammatory signals from DC during priming. In concert, these of stimulations determine the fate of T cell clonal expansion and differentiation. Greater proliferation correlates with stronger signal. However, insufficient stimulation results in death by neglect, while excessive stimulation causes activation induced cell death. Stronger stimulation also drives T cells toward terminal differentiation and reduces their memory-forming potential. Please see Section “Effector and Memory T Cell Differentiation Rules” for a description of differentiation models and how this was selected.

We based the cell differentiation process on a version of a “signal-strength model,” in which the overall strength of signal received by a naïve T cell during DC contact will determine the fate of cell differentiation (Figure 3) (32–35). A definitive differentiation scheme after T cell priming occurs has not been determined by experimentation. Previous modeling studies based on experimental data reject memory to effector differentiation in favor of effector to memory differentiation (20); however, more recent work showed that differentiation has as its backbone differentiation from naïve to CM precursor to EM precursor to effector (18). The scheme we use in this study considers effector to EM differentiation, but is still topologically similar to the scheme from (18), with precursors of both EM and effectors differentiating into these two subtypes (Figure 3). The difference between the two schemes is that “effectors” in our model are cells that have differentiated toward effector phenotype sufficiently so as not to enter into the CM population, nor have they entered into the EM pool. They are allowed to exit the LN due to the loss of early activation markers (CD69), even though these cells do not perform effector functions until they would reach sites of infection, which is not studied in this current work.

In our model, a series of probabilistic checkpoints are established to determine to which state a cell will proceed (36–39). When a cognate T cell finds an Ag-bearing DC (Ag-DC) or licensed DC (LDC) in its binding area, the corresponding pMHC value of the DC is checked to see if a successful binding can be established. If bound, a T cell continuously accumulates signals from the DC (40), represented by pMHC levels at each time point. Here, pMHC level is used as a proxy for the strength of antigenic stimulation from the DC or LDC. When a T cell unbinds from a DC or LDC, the accumulated signal value is used to determine whether a T cell proceeds to an activated state, or returns to a resting state (naïve). Activated cells go through a set number of rounds of divisions, after which the accumulated signal level is checked again to decide if the cell can further differentiate into an effector state. Effector cells will divide a few more rounds. With given probabilities, the cells with intermediate differentiation status do not proceed to effector status, but become CM cells, while those effector cells with sufficient signals will become EM cells (41–43). The probability of effector cell converting to EM is estimated between 0.1 and 0.4. CM T cells can be recruited to LNs from HEVs. These cells act similarly to cognate naïve T cells. When they detect Ag-DCs or LDCs, CMs will bind to DC and accumulate signal more efficiently in comparison with naïve cells (44, 45). The rules above apply to both CD4+ and CD8+ T cells. Because we developed some of these rules based on LCMV studies, one difference we captured between these two cell types is that CD8+ T cells can bind only to LDCs to generate functional memory cells in the primary response, whereas CD4+ T cells do not have this restriction and can generate memory cells after binding to both Ag-DCs and LDCs (46).

Other models of T cell differentiation exist, and some of these models are not mutually exclusive. We also integrated features from these models into our rule set, and excluded those that are inconsistent with current findings or are not applicable to our model at this stage. A single naïve T cell can produce both effector and memory progenies (19, 47), so we excluded the possibility that effector and memory arise from separate precursors. In the decreasing-potential model (13), the stimulation that T cells receive during infection drives greater clonal expansion but reduces their potential to differentiate into memory cells. Some studies show T cells are committed to massive proliferation after initial encounters with APCs, and can differentiate into both memory and effector subsets even if adoptively transferred into hosts absent of antigen (48). Thus, we limited the signal accumulation stage to the period of time when a T cell is bound to a DC before its first division, similar to findings made in B cell expansion (49). In the asymmetric cell fate model (50), heterogeneity arises from the unequal distribution of differentiation factors into daughter cells during division. We will further study this hypothesis as we incorporate dynamics at molecular level, but currently account for these asymmetries using phenomenological probabilities.

### Blood compartment sub-model: ODE and parameter estimation

We developed a blood compartment model by assuming the blood is a well-mixed, homogenous compartment. We use a system of non-linear ODEs to capture the dynamics of T cells therein. Equations for CD4+ T cells are:

(1) $$\begin{array}{ll} \frac{dN_{4}}{dt} & =S_{N4}\left(t\right)-\delta_{N4}N_{4}+e_{N4}^{\mathrm{LN}} \end{array}$$

(2) $$\begin{array}{ll} \frac{dE_{4}}{dt} & =-\delta_{E4}E_{4}-\xi_{E4}E_{4}+e_{E4}^{\mathrm{LN}} \end{array}$$

(3) $$\begin{array}{ll} \frac{dCM_{4}}{dt} & =-\delta_{\mathrm{CM}4}CM_{4}+\alpha_{\mathrm{EM}4}EM_{4}+e_{\mathrm{EM4}}^{\mathrm{LN}} \end{array}$$

(4) $$\begin{array}{ll} \frac{dEM_{4}}{dt} & =-\delta_{\mathrm{EM4}}EM_{4}-\xi_{\mathrm{EM}4}EM_{4}-\alpha_{\mathrm{EM}4}EM_{4}+e_{\mathrm{EM4}}^{\mathrm{LN}} \end{array}$$

*N*_4_, *E*_4_, CM_4_, and EM_4_ represent the blood concentrations of naïve, effector, CM, and EM CD4+ T cells, respectively. *S_N_*_4_(*t*) is the time-dependent thymus output of naïve CD4+ T cells (51). The initial output is estimated from healthy 30-year-old individuals, and declines by 5% per year (52). δ*_N_*_4_ is the overall death rate constant for naïve cells, including homeostatic proliferation and death. We estimated this parameter by assuming a quasi-equilibrium between thymus output and peripheral loss. δ*_E_*_4_, δ_CM4_, and δ_EM4_ are the death rate constants for effector, CM, and EM CD4+ T cells, respectively. δ*_E_*_4_ and δ_EM4_ account for the death of circulating effector and EM cells, excluding those recruited to sites of infection (53). δ_CM4_ reflects the overall loss of CM cells, including self-renewal and death (53). ξ*_E_*_4_ and ξ_EM4_ are the rate constants for recruitment of CD4+ effectors and EM cells from blood to sites of infection. As the dynamics at a site of infection are not considered in this study, these recruitment terms serve as a sink for the corresponding cell species in the blood compartment. α_EM4_ is the rate constant for EM cell differentiation into CM cells (54). The terms $e_{N4}^{\mathrm{LN}},\;e_{E4}^{\mathrm{LN}},\;e_{\mathrm{CM}4}^{\mathrm{LN}},$ and $e_{\mathrm{EM}4}^{\mathrm{LN}}$ represent rates of LN net output of corresponding cells. These terms are converted to the changes in concentration in the blood per time step. For naïve and CM cells, this is calculated as the difference between the number of exited and recruited cells. For effector and EM cells, this is calculated as the number of exited cells. These four terms are not solved directly in the ODE system but rather are added as an initial condition before each blood time step is processed in the computational model. We show them in the equations for completeness. Similar equations and parameter estimates are written for CD8+ T cells (see Supplementary Material). Because the CM CD8+ T cells population is maintained for life, we assume a very small value for the loss rate constant δ_CM8_, corresponding to half-life of 20 years (53). See Table S3 in Supplementary Material for a complete list of parameters, definitions, values, units, and source references.

### Two-compartment hybrid model

**Figure 4:**
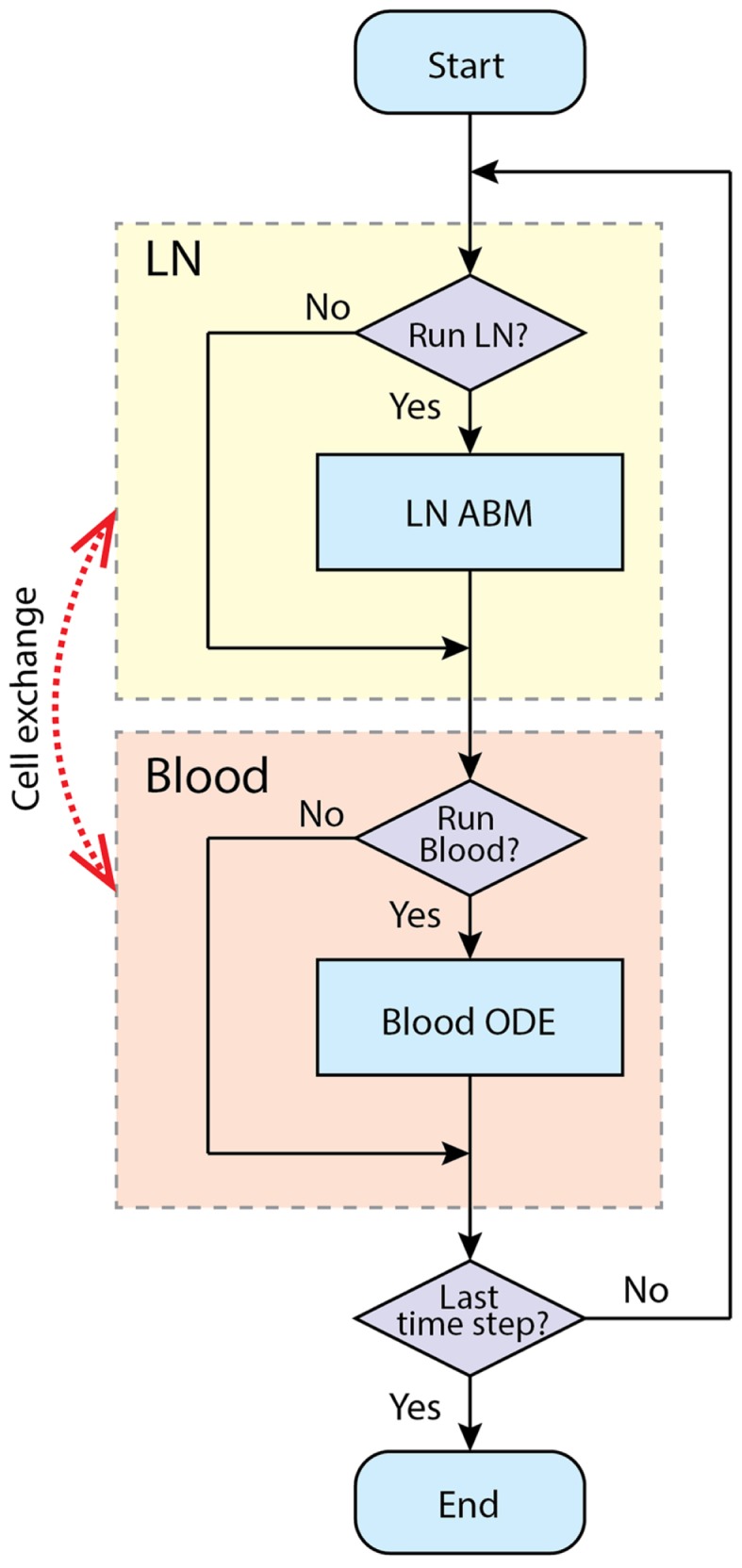
**Simulation procedure using tunable resolution**. LN and blood compartment are processed sequentially in each time step. LNs recruit cells from blood and put exiting cells into it. The recruitment probabilities are modified by blood concentration of corresponding cell types. Each compartment has a switch to determine whether this compartment is processed in current time step, or will it be bypassed.

Our goal is to develop a two-compartment computational model that combines *LymphSim* and the blood ODE model described above. Recently, we published other models linking ODEs and ABMs (55–57). For this study, we use the implementation method we employed successfully to link a LN compartment with a lung (56). The LN and blood compartment models are processed sequentially during each time step of simulation (Figure 4). During the T cell recruitment subroutine of the LN ABM model, the probability of recruiting T cells of each type/state is calculated based on their blood concentration levels. At the end of LN compartment simulation time step, the LN net output is calculated as the difference between exited and recruited number of each cell type and is multiplied by a factor that accounts for physiological compartment-size scaling from 0.5% back to the entire paracortex and unit conversion from cell number to blood concentration. This net output is then added to the corresponding variables in blood compartment ODEs. We have made a few assumptions regarding how we capture the LN to blood dynamics. First, we are only modeling dynamics of T cells and DCs within a single LN. There are ~700 LNs in the human body and they are connected via an intricate lymphoreticular network. T cells travel between multiple LNs via these lymphatics and eventually enter the blood via the superior vena cava. We assume that cells exit the LN and enter the blood compartment immediately, coarse-graining the time spent in the lymphatic system. However, our cells travel through the LN and blood in time frames consistent with experimental data [<24 h; Ref. (58)], accounting for the delay.

For computational efficiency, we use a method we term *tunable resolution* (TR) (manuscript submitted). One of the goals of TR is to develop multi-scale models with sub-models of different resolutions, so that models can be run with coarse- or fine-grained alternative versions of sub-models during simulation to save resources without sacrificing accuracy. Here, for each physiological compartment (blood or LN), there is a computational switch that allows the model in an automated fashion to bypass simulation of a given compartment. In this two-compartment model, we do not have an alternate version of each compartment *per se*; instead, each compartment can be suspended when specific criteria are met. For example, during the pre-simulation, the blood compartment is turned off, and the LN is simulated until a baseline steady state is reached. When an immune response is occurring, LN and blood compartments are both running to simulate the immune response in fine-grained, spatially explicit detail for a time scale of a few weeks. When an active immune response finishes and there are no Ag-DCs, LDCs, bound, active, effector, or EM cells in the LN compartment, the LN compartment is suspended to allow rapid simulation of the blood compartment at longer time scales (months to years). When a secondary infection begins, the LN compartment is switched on again (Figure 4).

### Model calibration

The hybrid model contains 103 parameters that govern mechanisms occurring in both physiological compartments and the interactions between them (see Table S3 in Supplementary Material for a complete list of the parameters). For the LN compartment model, parameters governing T cell motility and trafficking are calibrated to data as described previously (30). Parameter estimates for the ODE model in the blood compartment are discussed in Section “Blood Compartment Sub-Model: ODE and Parameter Estimation” and Supplementary Material.

**Figure 5:**
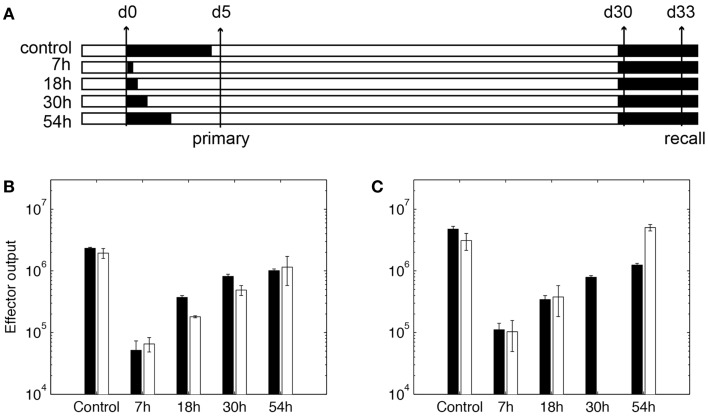
**Expansion of CD8+ T cells in simulation**. **(A)** *In silico* experimental schemes. Black bars show the duration of Ag-DC presence. In primary challenge, DC antigen presentation is terminated at time points indicated on the left. “Control” indicates no termination and DC are allowed to live their natural lifespan. Recall challenge is given from day 30. Measurements are taken on day 5 for primary response and day 33 for recall response, respectively. **(B,C)** CD8+ T cell population in simulated responses (black bars) and experimental data (white bars) (59). **(B)** Size of expanded CD8+ T cell population in primary response. Values are measured from day 5 after Ag-DC are recruited to the LN. **(C)** Size of expanded CD8+ T cell population during recall response on day 33. *X*-axis value indicates antigen presentation times in the primary challenge.

To use our model for memory T cell differentiation dynamics, we estimated parameters in our model using the limited data available in the literature for memory cell generation in LNs. We estimated parameters governing total production of expanded cognate CD8+ cells generated in the LN model (Table S3 in Supplementary Material, parameters marked with ‡) to data from T cell clonal expansion studies in mice using OVA as a stimulating antigen (59). In that study, DCs are ablated at different time points to show that the duration of antigen presentation correlates with magnitude of T cell expansion, but a short exposure is sufficient to program CD8+ T cells to differentiate into both effector and memory subsets (59). We adapted our model to reflect experimental methods used in these studies. DCs are removed from the LN grid at indicated time points after the recruitment during primary challenge (Figure 5A), as was done experimentally by injecting diptheria toxin (DT) (59). Unlike rules for LCMV as previously discussed, CD8+ naïve T cells are allowed to bind both Ag-DC and LDC to be primed and the enter memory state. This is because in these experiments, DCs are activated from LPS pulsing or *Listeria monocytogenes*-OVA. From our *in silico* experiments terminating antigen presentation from DCs at various time points after Ag-DC recruitment, we predict that the magnitude of the primary response is dependent of the duration of DC presence (Figure 5B). However, a very short period of stimulation is capable of generating memory cells, as we see a potent production of antigen-specific CD8+ T cells after a secondary challenge (Figure 5C). Moreover, it takes only 3 days for the recall response to exceed the magnitude of primary response on day 5, indicating a faster reaction to previously experienced antigens, as observed *in vivo*. Our simulation results are comparable to data from the Prlic study (59). The parameter set we obtained is used as our baseline for simulating infection scenarios (see below and Table S3 in Supplementary Material).

### Simulated infection and model validation

We next validated our model with data sets from experimental studies using LCMV or OVA as stimulating antigens. For each simulated infection, a 3-day pre-simulation of the ABM LN sub-model precedes the actual experiment to allow cells to reach a steady state in terms of quantity and spatial distribution. During this period, the blood sub-model is suspended, with the naïve CD4+ and CD8+ T cells concentrations fixed at 450 and 320/mm^3^, respectively (51, 60). Then Ag-DCs are introduced to stimulate the T cell response. We represent this by introducing antigen-bearing DCs in such a way as to mimic an acute infection (23, 25, 30). Ag-DCs carry and present a unique antigen and are recruited to the LN compartment for 2 days. These DCs will prime cognate T cells (cognate frequency is set to 10^−4^) for about 5 days before they die, mimicking a hypothetical acute infection. To mimic a hypothetical secondary infection, Ag-DCs are recruited to the LN again from day 600 to 602. Each experiment is simulated five times to reduce aleatory uncertainty.

To confirm that our model produces reasonable dynamics in the blood compartment, we qualitatively compare the time course of blood antigen-specific cells to data sets from LCMV studies, where the measurements are performed in spleen (61–63).

For lineage tracing simulations (see Cognate Naïve Cells Undergo Heterogeneous Expansion), every cognate naïve T cell recruited to the LN is assigned a unique serial number. This number is passed to the daughter cells when these labeled T cells proliferate and differentiate, so T cells sharing the same serial number belong to the same single-cell derived progeny. When each differentiated cell exits the LN, its serial number is recorded. For each individual cognate precursor, the number of descendant cells and their differentiation states are calculated. Cell progenies are ranked by the abundance of their progenies, from largest to smallest. Five replications are performed for this simulation.

We calculate the Index of disparity *D* between the expanded populations of different single-cell derived progenies (19), which is the inverse Simpson diversity index mapped to a 0–1 interval:

(5) $$D=\frac{N-\frac{1}{\sum_{i=1}^{N}f_{i}^{2}}}{N-1},$$

where *N* is the number of progenies and *f* is the frequency of each single-cell derived progeny in the total population.

### Uncertainty and sensitivity analysis

In this study, our goal is to reproduce patterns of generalized immune responses. In addition to using parameters estimated from previous work (see above and Table S3 in Supplementary Material) and experimental data, we use global uncertainty and sensitivity analysis (U/SA) to study how particular biological mechanisms affect simulation outputs (64).

For each set of sensitivity analysis, a list of parameters is chosen, and for each parameter of interest, a range is specified. Latin hypercube sampling (LHS) is applied to generate the matrix of parameter values, where each experiment represents one combination of sampled parameter values. LHS is a stratified sampling method that requires fewer samples compared with random sampling method but achieves the same accuracy (65). This technique is particularly helpful for our ABM model where parameter values need to be estimated from a high-dimensional space (64). The parameter space is sampled completely and accurately, with a large sampling size. Each experiment is replicated five times to reduce aleatory uncertainty from inherent stochastic variations (64). After the simulation, model readouts are chosen and partial rank correlation coefficients (PRCCs) are calculated between each readout-parameter pair to assess global sensitivity and detect monotonic relationship between mechanisms and output of interests.

To study how various mechanisms affect the generation of memory from within each compartment (blood and LN) as well as how they influence the other compartment (LN and blood, respectively), we performed intra- and inter-compartment sensitivity analysis (64). We choose two sets of parameters governing mechanisms in each sub-model and estimated a range for each parameter (see Table S3 in Supplementary Material). We use LHS to sample the parameter space and generate 100 or 408 experiments for blood and LN experiments, respectively. Here we performed 2540 simulations, which provides ample coverage of the space. Sensitivities of outputs to mechanisms are assessed with PRCCs.

### Computational simulations and implementation

Our hybrid model is implemented in C++ and runs on Linux/Mac OS/windows. Documentation and pseudo code are available in the online Supplement. A Forward Euler method is used to solve the ODEs. Each time step of the ABM simulation is further divided into 100 pieces (step size of 0.25 s) to reduce error. Each simulation of 350 days (LN sub-model is active for ~40 days) takes 30–40 h to run.

## Results

### Healthy uninfected baseline dynamics of T cells are reached without simulated antigen presentation

Without Ag-DC introduced to the LN, all T cells remain naïve. The cell dynamics in LN in the absence of any infection present show that over a short time scale (days to weeks), cells remain at the steady state of ~170,000, or 4.0 × 10^6^ cells/mm^3^. There is an equal input/output flow of ~1000 cells per million cells per minute, and an average transit time of 16 h, which is consistent with previous data (30). The population of both CD4+ and CD8+ T cells in the blood declines long-term (20 years). By the end of year 20, the blood concentration of naïve CD4+ T cells drops to 210 mm^−3^, and that of naïve CD8+ T cell drops to 170 mm^−3^. Such long-term decline of naïve T cell number is comparable to clinical observations (66, 67).

### Effector and memory T cell populations are generated in a simulated acute infection

**Figure 6:**
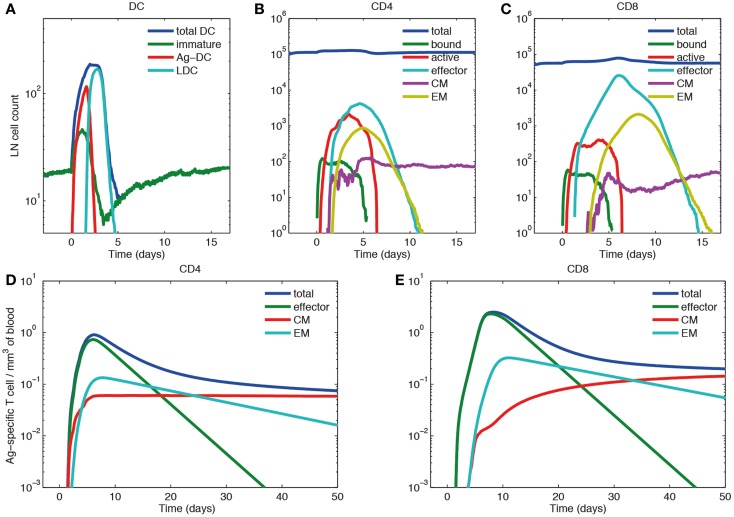
**Primary response dynamics of immune cells in LN and blood during a hypothetical acute infection (log scale)**. **(A–C)** Number of dendritic cells, CD4+ and CD8+ T cells of different subsets in the LN compartment. **(D,E)** Concentration of CD4+ and CD8+ T cells of different subsets in the blood compartment. **(A)** Model input of DCs representing a hypothetical acute infection, such as LCMV and **(B–E)** output.

We simulated immune responses to a hypothetical acute infection by introducing Ag-DCs into the LN compartment to activate cognate T cells as shown in Figure 6A. The cognate frequency is set to 10^−4^. Figures 6B–E show simulated immune cell dynamics in the LN and blood compartments.

In the LN compartment, the Ag-DC population increases first. These DCs scan surrounding CD4+ and CD8+ T cells and bind to their cognate matches. After this binding event, CD4+ and CD8+ T cells begin to proliferate and differentiate into active and effector T cells. After day 2, the influx of Ag-DCs to LN stops, and the number of Ag-DCs begins to decline (Figure 6A). At the same time, differentiated effector CD4+ T cells license Ag-DCs, further increasing their surface pMHC levels and stimulation strength, enabling them to allow CD8+ T cell memory potential. Because we assume that CD8+ T cell memory establishment requires LDCs, the appearance of CM and EM CD8+ T cells is delayed as compared with corresponding CD4+ T cells. After differentiation from the active state, effector, CM, and EM cells can exit the LN from ELs, resulting in the decline of these populations within the LN. The system eventually returns to baseline, but CMs can still recirculate through LN (Figures 6B,C).

In the blood compartment, the concentrations of effector, CM, and EM cell populations increase as they exit the LN (Figures 6D,E). The total concentration of both CD4+ and CD8+ Ag-specific T cell (effector, EM, and CM) peaks at about day 6 and 8, respectively (0.91 mm^−3^ for CD4+ T cells and 2.49 mm^−3^ for CD8+ T cells). The lifespans of effector cells are relatively short. These cells either die, or are recruited to sites of infection, bringing about a contraction phase characterized by a decline of total blood Ag-specific T cells. However, about 5% of the peak level is maintained in the memory cell class, especially CM cells in the long-term as their lifespan is longer than EM cells. In the blood, some of the EM cells convert to CM cells, while others are recruited away to sites of infection (Figures 6D,E). While there are no data from primates on these dynamics, our results are qualitatively in accordance with experimental data from mouse LCMV studies (61–63).

### Immune cells reach higher levels during a recall response as compared to a primary response

To understand the dynamics of a recall response, we simulated a scenario where Ag-DCs are introduced from day 0 to 2 in an initial round of infection (the same as that of Section “Effector and Memory T Cell Populations are Generated in a Simulated Acute Infection”). Once that infection dampens and immune cells return to a resting state, we introduce a second round of challenge by recruiting Ag-DCs from day 600 to 602. We challenge with the same antigen and use the same cognate frequency for naïve cells, but CM populations are maintained in the blood after the primary response. The resulting dynamics of Ag-specific T cells occurring in blood are shown in Figure 6.

**Figure 7:**
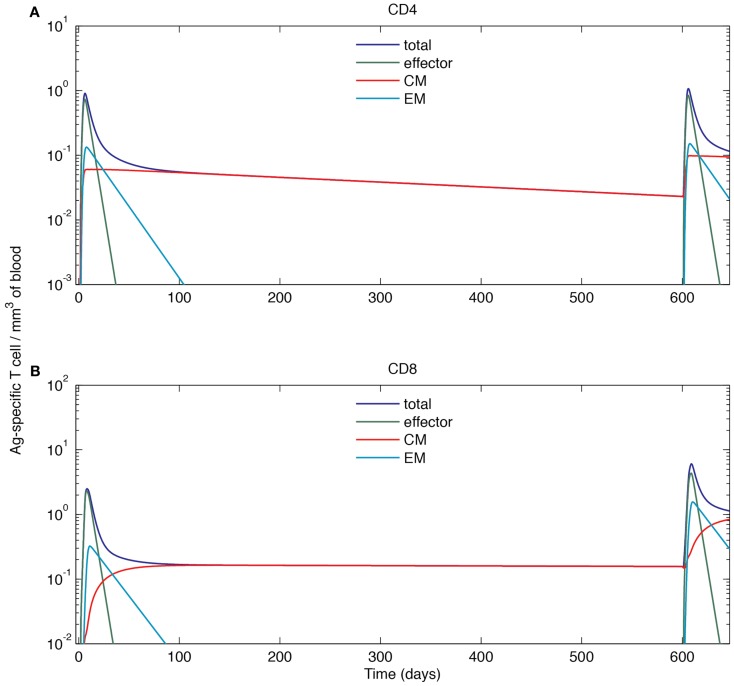
**Simulated cell dynamics in the blood compartment during a primary and recall response to a hypothetical acute infection (log scale)**. **(A)** Concentration of CD4+ T cells of different subsets in the blood. **(B)** Concentration of CD8+ T cells of different subsets in the blood. The left parts of the graphs are identical to those of Figure 6.

As above, the primary response is initiated after the first round of Ag-DC input. Blood Ag-specific T cell numbers rise as the response continues and peak at day 6 and 8. After the peak, effector and EM T cells decline while the CM cell population is maintained. On day 600, the blood concentration of CM CD4+ T cells has dropped from 0.059 to 0.023 mm^−3^, while the CM CD8+ T cell population remains at 0.16 mm^−3^. The stable maintenance of CD8+ memory and decline of CD4+ memory is in agreement with mouse LCMV infection data (53). During the recall response, because of a memory cell population generated during the primary response that can faster and more strongly respond to the same antigen, both CD4+ and CD8+ T cells in the blood exceed peak levels of their primary response, peaking at 1.07 mm^−3^ for CD4+ and 6.05 mm^−3^ for CD8+ T cells. The recall response is more than twice as large as primary response for CD8+ T cells, but only marginally increased (18%) for CD4+ T cells. Such differences in CD4+ and CD8+ recall responses have been observed in LCMV experiments as well (68). After the recall response, higher levels of CM cells are maintained as compared to following the primary response (Figure 7). After the recall response ceases, the blood concentrations of CM cells are 0.094 and 0.84 mm^−3^ for CD4+ and CD8+ T cells, respectively. These results indicate that the antigen-specific immune memory is reinforced after the second round of challenge, as the central memory population formed in the primary challenge gets further expanded during the second round of challenge.

### Cognate naïve cells undergo heterogeneous expansion

Recent lineage tracing studies showed that CD8+ T cells have a heterogeneous differentiation pattern (18, 19). Because the LN compartment of our model is agent-based, it is possible to track the fate of each individual cell during a simulated infection. We take advantage of this feature to validate our model using data from these recent studies.

**Figure 8:**
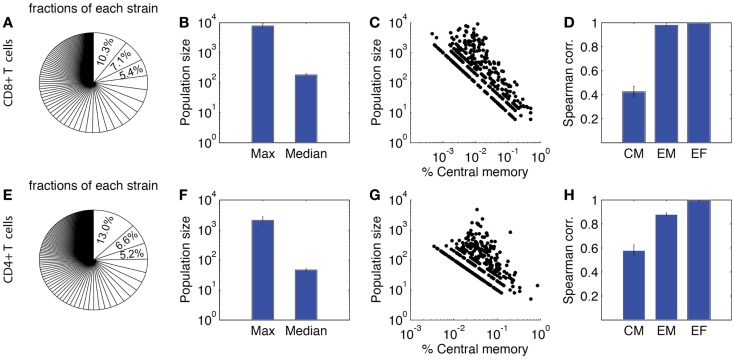
**Heterogeneity of expanded T cell families**. Upper panel: CD8+ T cells. **(A)** Sizes of expanded CD4+ T cell population from each single-cell derived progeny (strain), represented as fraction of total expanded population as done in Buchholz et al. (18) and Gerlach et al. (19). **(B)** Maximum and median size of CD4+ T cell progenies. **(C)** Correlation between population size and the percentage of central memory cells. Each dot represents progeny of a single-cell derived progeny. **(D)** Spearman correlation of CM, EM, and effector CD4+ T cell number versus total expanded population. **(E–H)** Prediction of CD4+ T cell heterogeneity in clonal expansion. The four panels correspond to the same readouts as for panels **A–D**.

Figure 8 shows our lineage tracing analysis for the primary response. The fraction of each single-cell derived progeny in the total population is shown in Figure 8A for CD8+ and Figure 8E for CD4+ T cells. For both CD4+ and CD8+ T cells, a small number of progenies have a large expanded population. The average size of the largest population is ~2000 for CD4+ T cells and ~8000 for CD8+ T cells. However, the majority of derived progenies have intermediate to small population sizes, with about 50 for CD4+ T cells and 200 for CD8+ T cells. The maximum population size of CD8+ T cell is 50-fold larger than the median. The index of disparity in our simulations is 0.81 for CD8+ T cells, close to the range of 0.85–0.95 shown in Ref. (19). These results indicate our model matches well with the heterogeneous differentiation experimental observations. While the corresponding experimental studies were performed only for CD8+ T cells, we are able to use our model to simulate the dynamics of CD4+ T cells as well. Our model predicts less heterogeneity for CD4+ T cells, with an index of disparity of 0.82 and a 50-fold difference between largest and median progenies (Figure 8F).

We also assessed the composition of these sub-populations. In Figures 8C,G, the proportion of CM cells of each single-cell derived progeny is plotted against the population size. These results suggest that progenies with a higher proportion of CM cells tend to have a smaller expanded population during the primary response. We calculated the Spearman correlation coefficients between each subtype and the total number of expanded cells (Figures 8D,H). The correlations are strong between effectors and overall total population size, but weak between CM and the overall population size. This is comparable to the results from Ref. (18). Thus, in addition to our other findings, these results confirm those previously identified (18, 19) that the magnitude of the primary response for single-cell derived progenies might not be the sole predictor of immune memory. We next study, which mechanisms influence the heterogeneous differentiation and clonal expansion processes of T cells.

### Antigen presentation by DCs influences outcome of an immune response

**Figure 9:**
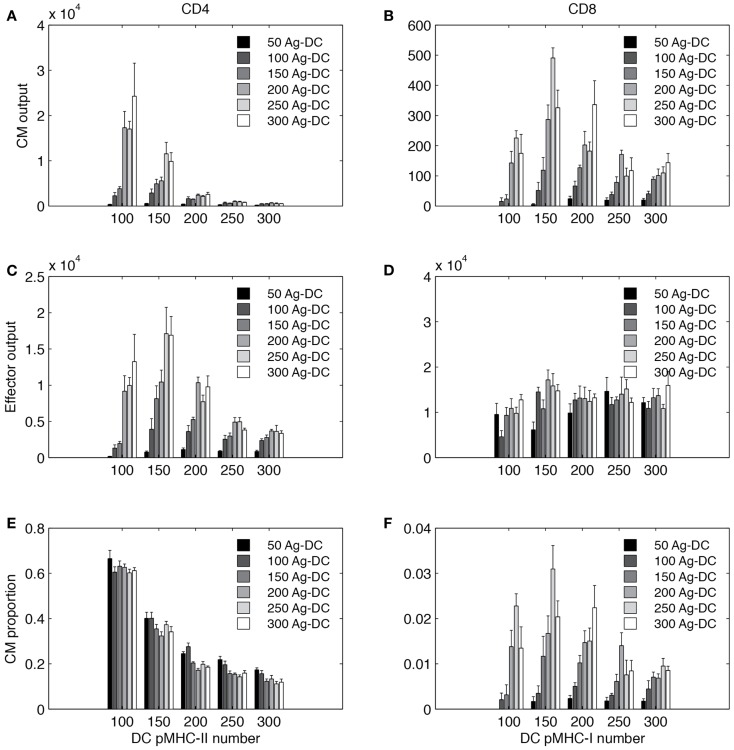
**Simulated T cell differentiation is influenced by number of DCs and number of pMHC molecules displayed**. *X*-axis: number of pMHC molecules on an Ag-DC. Bar color: number of Ag-DCs recruited to the grid. **(A,B)** Number of central memory (CM) cells produced. **(C,D)** Number of effector cells produced. **(E,F)** Fraction of CM cells in the expanded population.

It is no surprise that antigen stimulation plays a crucial role in T cell activation and differentiation (35, 69). However, it is difficult to conduct experiments that quantitatively determine the mechanism of dependency. Here we varied the number of Ag-DCs recruited to LN in a range of 50–300 and the levels of pMHC molecules on the surface Ag-DCs from 100 to 300, and analyzed how they influence production of effector and CM cells. Model pMHC levels are used as a proxy for DC stimulation strength. Results are shown in Figure 9.

Increasing the number of Ag-DC recruitment promotes the output of both effector and memory T cells from the LN. The number of Ag-DC has a larger impact when the pMHC molecule levels are low. This result indicates that more Ag-DCs are beneficial for the production of higher levels of effector and memory cells, but this benefit is diminished when pMHC molecule levels are high.

Interestingly, for each subset of cells, the effects of increased pMHC molecule levels on the surface of DCs are different. pMHC levels are always negatively correlated with CM output of CD4+ T cells (Figure 9A). However, the highest numbers of effector CD4+ T cell output are reached at intermediate levels of pMHC (Figure 9C). CD8+ T cells are affected in a different pattern. Intermediate levels of pMHC are required for higher CM production (Figure 9B). Our explanation for this difference is that we defined our rules based on LCMV experiments and other studies suggesting that CD8+ T cell memory establishment is dependent on DC licensing by effector CD4+ T cells, and intermediate levels of pMHC are required so that LDC numbers will not be the bottleneck for memory CD8+ T cell production. Also different than CD4+ T cells, effector CD8+ T cell output first increases with pMHC levels on DCs and then remains relatively stable (Figure 9D). The fraction of CM among total expanded population is shown in Figures 9E,F.

In general, our simulations suggest that in order to obtain high CD4+ T CM cell production, DCs with lower pMHC levels should be provided. However, DCs with high pMHC levels maximize CD8+ effector output. CD4+ effectors and CD8+ CM T cells require intermediate pMHC levels. Increased recruitment of Ag-DC boosts all four subsets to different extents.

### Sensitivity analysis detects mechanisms correlated with strength of recall responses

We also studied how other mechanisms, such as thresholds of different checkpoints in the LN, conversion rates of EM to CM, and recruitment rate to sites of infection from the blood, shape model outputs (see Table S3 in Supplementary Material for parameters varied. The mechanisms they control are explained in the column “description”). We performed intra- and inter-compartment sensitivity analysis (see Materials and Methods), and PRCCs were calculated to assess the monotonic correlation between values of the parameters governing these mechanisms, and outputs including: LN production and blood concentration of effector and CM cells in both primary and recall responses (Tables 1–3).

**Table 1:** **PRCC results: tracking sensitivity of outputs of LN cells to LN mechanisms**.

Primary			Recall		
Mechanism	PRCC	Significance	Mechanism	PRCC	Significance
**CD4+ EFFECTOR GENERATED FROM LN**					
Bind time	−0.90	−−−	Bind time	−0.82	−−−
Probability EM	−0.73	−−−	Probability EM	−0.64	−−−
Priming checkpoint threshold	−0.42	−−−	Priming checkpoint threshold	−0.40	−−−
CM bind time	−0.29	−−	CM bind time	−0.37	−−−
DC licensing probability	−0.26	−−	DC licensing probability	−0.29	−−
Extra recruitment	0.38	+++	Efficiency CM	−0.27	−−
Effector checkpoint threshold	0.67	+++	Extra recruitment	0.35	+++
			Effector checkpoint threshold	0.61	+++
**CD4+ CM GENERATED FROM LN**					
Bind time	−0.88	−−−	Bind time	−0.62	−−−
Priming checkpoint threshold	−0.70	−−−	Priming checkpoint threshold	−0.48	−−−
Efficiency CM	−0.29	−−	CM bind time	−0.44	−−−
CM bind time	−0.26	−−	Efficiency CM	−0.33	−−−
Extra recruitment	0.33	+++	Extra recruitment	0.26	++
Effector checkpoint threshold	0.81	+++	Effector checkpoint threshold	0.61	+++
**CD8+ EFFECTOR GENERATED FROM LN**					
Extra recruitment (CD4)	−0.56	−−−	Probability EM	−0.74	−−−
Bind time	−0.41	−−−	Extra recruitment (CD4)	−0.56	−−−
DC licensing prob.	−0.25	−−	Bind time (CD8)	−0.37	−−−
Probability EM	−0.25	−−	DC licensing probability	−0.21	−−
**CD8+ CM GENERATED FROM LN**					
Bind time	−0.71	−−−	Efficiency CM	−0.42	−−−
Priming checkpoint threshold	−0.71	−−−	CM bind time	−0.35	−−−
CM bind time	−0.19	−	Priming checkpoint threshold	−0.22	−
Extra recruitment	0.30	++	Bind time	−0.18	−
Effector checkpoint threshold	0.77	+++	Prob. EM	0.33	+++
			Effector checkpoint threshold	0.35	+++

*−/+, *p* < 0.001, with negative or positive correlation; −−/++, *p* < 10^−6^, with negative or positive correlation; −−−/+++, *p* < 10^−9^, with negative or positive correlation*.

We found that production of effector cells in primary responses is most sensitive to the following mechanisms: binding time (negatively correlated, Tables 1 and 2), the probability of effectors differentiating into EM (negatively correlated, Tables 1 and 2), and extra recruitment in inflammation (positively correlated, Tables 1 and 2). In addition, CD4+ T cell effector output negatively correlates with the stimulation threshold for priming but positively correlates with the threshold for differentiation into effectors (Tables 1 and 2); CD8+ T cell effector cell output negatively correlates with extra recruitment of CD4+ T cells (Tables 1 and 2). Generation of CM cells during a primary response is sensitive to a similar set of mechanisms, along with some additional ones: CD4+ T cell CM output is negatively affected by the efficiency of CM cells to accumulate stimulation signal; CD8+ T cell CM output is positively correlated with threshold to become effectors (Tables 1 and 2). Interestingly, in the recall response, the mechanisms to which effector cell production are sensitive are consistent with that of those affecting effector cells and CM cells during primary responses. For instance, the LN output of effectors in the recall response has a more significant negative correlation with EM differentiation probability than in the primary response, blood CM concentration. This is in accordance with the intuition that a strong recall response requires both memory generation during a primary response and priming efficiency during secondary challenge.

**Table 2:** **PRCC results: tracking sensitivity of concentrations of cells in Blood to LN mechanisms**.

Primary			Recall		
Mechanism	PRCC	Significance	Mechanism	PRCC	Significance
**CD4+ EFFECTOR CONCENTRATION**					
Bind time	−0.83	−−−	Bind time	−0.71	−−−
Probability EM	−0.74	−−−	Probability EM	−0.67	−−−
Priming checkpoint threshold	−0.42	−−−	DC licensing probability	−0.39	−−−
DC licensing probability	−0.41	−−−	CM bind time	−0.38	−−−
CM bind time	−0.29	−−	Priming checkpoint threshold	−0.35	−−−
Extra recruitment	0.32	+++	Efficiency CM	−0.25	−−
Effector checkpoint threshold	0.64	+++	Extra recruitment	0.27	++
			Effector checkpoint threshold	0.53	+++
**CD4+ CM CONCENTRATION**					
Bind time	−0.89	−−−	Bind time	−0.70	−−−
Priming checkpoint threshold	−0.71	−−−	Priming checkpoint threshold	−0.53	−−−
Efficiency CM	−0.30	−−	CM bind time	−0.43	−−−
CM bind time	−0.26	−−	Efficiency CM	−0.33	−−−
Extra recruitment	0.33	+++	Extra recruitment	0.26	++
Effector checkpoint threshold	0.81	+++	Effector checkpoint threshold	0.66	+++
**CD8+ EFFECTOR CONCENTRATION**					
Extra recruitment (CD4+)	−0.57	−−−	Probability EM	−0.72	−−−
Bind time	−0.31	−−−	Extra recruitment (CD4+)	−0.55	−−−
DC licensing probability	−0.28	−−	Bind time (CD8+)	−0.28	−−
Probability EM	−0.27	−−	DC licensing probability	−0.23	−
			Bind time (CD4+)	0.18	+
**CD8+ CM CONCENTRATION**					
Probability EM	−0.78	−−−	Probability EM	−0.82	−−−
Bind time	−0.53	−−−	Extra recruitment (CD4+)	−0.42	−−−
Priming checkpoint threshold	−0.39	−−−	Bind time	−0.38	−−−
Extra recruitment (CD4+)	−0.37	−−−	Efficiency CM	−0.30	−−
Effector checkpoint threshold	0.52	+++	CM bind time	−0.27	−−
			Priming checkpoint threshold	−0.25	−−
			Effector checkpoint threshold	0.39	+++

*−/+, *p* < 0.001, with negative or positive correlation; −−/++, *p* < 10^−6^, with negative or positive correlation; −−−/+++, *p* < 10^−9^, with negative or positive correlation*.

We then perform an inter-compartment sensitivity analysis by comparing how the readouts for the same cell types from both LN and blood are affected by corresponding LN and blood mechanisms. LN mechanisms affect both LN and blood outputs in similar ways, but only blood outputs are sensitive to blood mechanisms (Table 3). The recruitment rates of effector cells to sites of infection reduce blood effector levels. Conversion rates from EM to CM induce both blood CD4+ and CD8+ T cell CM levels. Also, our predictions suggest that dynamics occurring in blood do not significantly affect dynamics occurring in LNs during both primary or recall responses, as no significant correlation is detected.

**Table 3:** **PRCC results: tracking sensitivity of concentrations of cells in blood to blood mechanisms**.

Primary			Recall		
Mechanism	PRCC	Significance	Mechanism	PRCC	Significance
**CD4+ EFFECTOR CONCENTRATION**					
Recruit to site of infection (ξ*_E_*_4_)	−0.79	−−−	Recruit to site of infection (ξ*_E_*_4_)	−0.54	−−
CD4+ CM
Recruit to site of infection (ξ_EM4_)	−0.39	−	Probability EM	0.44	+
Probability EM	−0.80	+++			
**CD8+ EFFECTOR CONCENTRATION**					
Recruit to site of infection (ξ*_E_*_8_)	−0.39	−	Recruit to site of infection (ξ*_E_*_8_)	−0.43	−
			Recruit to site of infection (ξ_EM8_)	−0.36	−
			Probability EM	0.72	+++
**CD8+ CM CONCENTRATION**					
Recruit to site of infection (ξ_EM8_)	−0.41	−	Recruit to site of infection (ξ_EM8_)	−0.45	−
Probability EM	0.93	+++	Probability EM	0.83	+++

*−/+, *p* < 0.001, with negative or positive correlation; −−/++, *p* < 10^−6^, with negative or positive correlation; −−−/+++, *p* < 10^−9^, with negative or positive correlation*.

## Discussion

Single cognate naïve T cells have been known to be able to generate both memory and effector progenies. Moreover, recent studies further demonstrated that the fate of identical naïve cells is heterogeneous. By understanding which mechanisms contribute to this heterogeneity and in which way they are contributing, it is possible to manipulate the priming environment so that differentiation of activated precursor cells can be routed to favor generation of a desired population toward specific needs. For example, in the context of vaccination, establishing a significant antigen-specific CM population has a high priority. On the other hand, massive production of effector cells could be the key issue considered when using immunotherapies against active diseases. Our new findings suggest that pMHC number on the surface of APCs provides such a handle; and using our model we can enhance the production of specific T cell types (CD4+/CD8+, effector/memory) in different ideal ranges. By fitting our model to data collected from experiments designed for a specific antigen, we will be capable of making quantitative predictions of DC stimulation levels that maximize the generation of particular subsets of T cells, which are most relevant to the circumstances.

In order to study how mechanisms in LN and blood influence the generation of effector and memory T cells, we developed a hybrid model with both LN and blood compartments to simulate immune responses in both primary and recall challenges. Using this model, we generated T cell dynamics in blood and LN during infections that are similar to murine models (61–63) and also can capture heterogeneous differentiation observations of individual cognate naïve T cells (18, 19). Furthermore, our model predicts that the outputs of different subsets of T cells that arise during immune responses, including effector and memory, CD4+, and CD8+ T cells, respond differently to the amount of stimulation they receive from antigen-bearing DCs during priming. Simulations showed that CD4+ CM T cell generation is maximized at low pMHC-II levels, and intermediate pMHC-II levels result in the highest number of effector CD4+ T cell generation. However, further increases in pMHC-II levels reduce generation of both effector and CM CD4+ T cells. On the other hand, intermediate pMHC-I level is required to generate the highest levels of CD8+ CM cells, and high pMHC-I level favors CD8+ T effector cell generation.

Results from our previous study using a 2D model showed pMHC levels always compensate for DC numbers to induce effector T cell production (25) in a trade-off fashion. We find a similar trend herein for CD8+ T cells, and for CD4+ T cells, when DC numbers are small. But when DC numbers are large, high pMHC levels are playing an opposite (for CD4+ T cell) or insignificant (for CD8+ T cell) role. This can be explained by the findings from our 3D LN model (30), that DC searching time for T cells is far more efficient in a 3D model environment than 2D. Thus, even for high total DC numbers in the 2D study, there are likely insufficient DCs, suggesting what we observed in the 2D model represents only the case with relatively low DC numbers in 3D.

While our model is able to make some important predictions, further development to include more detail regarding events during antigen presentation is called for. First, DCs are known to be a heterogeneous population, with subsets of cells diversified in origin and function. Different DC subsets are differentially involved in T cell priming. For example, lymphoid organ-resident DCs are specialized for cross-presentation, while inflammatory DCs stimulate T_H_17 polarization (70, 71). Furthermore, the stimulation that T cells receive from DCs are also combinations of multiple signals, including TCR avidity, co-stimulation regulation, and environmental, such as inflammatory cytokine profiles. Reducing these signals to a general stimulation signal package represented with pMHC levels as done here helps conceptualize the question and generate theoretical insights; nonetheless, adding these details will confer power for predicting more precise manipulations of the immune response. Harnessing the power of both mathematical and computational modeling and wet-lab investigation, our systems biology approach can eventually provide guidance for clinical practices in an era of personalized medicine.

## Conflict of Interest Statement

The authors declare that the research was conducted in the absence of any commercial or financial relationships that could be construed as a potential conflict of interest.

## Supplementary Material

The Supplementary Material for this article can be found online at http://www.frontiersin.org/Journal/10.3389/fimmu.2014.00057/abstract
