# Supplementary material for: Harnessing the Heterogeneity of T Cell Differentiation Fate to Fine-Tune Generation of Effector and Memory T Cells
Source: Front Immunol. 2014 Feb 19;5:57. doi: 10.3389/fimmu.2014.00057 (PMC3928592; doi:10.3389/fimmu.2014.00057)
Supplement: Supplementary file 1 [file 64248_Kirschner_DataSheet1.DOCX]

**Supplemental for: Harnessing the heterogeneity of T cell differentiation fate to fine- tu­ne generation of effector and memory T cell­­­s**

**Supplemental Material**

**S1. Rules for ABM LN sub- model**

**S1.1 Environment**

**S1.1.1 Simulation setup**

Actual time per time step: 25 sec.

Size of a unit on grid: 5 microns.

Height of grid: 160 compartments.

Diameter of top slice: 70 compartments.

Diameter of bottom slice: 32.67 compartments.

**S1.1.2 LN Structure**

*i. AL*

Afferent lymph vessel: DCs enter from AL. Current setting (specified in parameter file): From slice 159 to slice 85, on the tip half of the lymph node. All the open space in this region sufficient to accommodate DCs before adding T cells is considered potential DC recruitment location.

*ii. HEV*

T cells are recruited from HEVs. Current setting (specified in parameter file): located from slice 154 to slice 50. Arranged into randomly generated clusters.

*iii. EL*

Efferent lymph vessel: T cells exit from EL. Current setting (specified in parameter file): Generated from slice 77 down. They are generated with higher probability to be in the vicinity of the edge of the grid (side or bottom) or near HEVs. There are 300 generated.

*iv. FRC network*

Generated by seeding at the edge of LN, then scan the entire grid for compartments with 1 or 2 FRC compartments in its Moore neighborhood. Set such compartments to FRC at probability of 0.1 and 0.01 (can be changed in parameter file). Repeat scanning for 100 times (should changed according to seeding position and grid size.)

**S1.2 Agents**

**S1.2.1 CD4 T Cells**

*i. Constants*

Naïve ecruitment probability: 0.00058 (mm^3^)

CM ecruitment probability: 0.0029 (mm^3^)

Extra recruitment: 1.25

ProbMoveInPreferredDirection: 0.85

ProbMoveInNonOppositeDirection: 0.95

Average naïve life: 365 (day)

Binding radius: 2

Median binding pMHC-II: 150

Binding curve shape: 15

Unbinding threshold: 100

Max naïve bind time: 12 (hour)

Max naïve bind time SD: 4 (hour)

Max CM bind time: 30 (min)

Max CM bind time SD: 10 (min)

Median priming pMHC-II: 1000 (hour)

Priming curve shape: 100 (hour)

Median effector pMHC-II: 2000 (hour)

Effector curve shape: 500 (hour)

Average active life: 96 (hour)

Average effector life: 60 (hour)

Average CM life: 365 (day)

Average EM life: 25 (day)

Min division: 4

Max division: 8

Doubling time: 6 (hour)

*ii. Recruitment*

Each HEV port tries to recruit one cell at every time step. The probability that a naïve or central memory (CM) CD4+ T Cell at every time step is calculated from the product of concentration of blood naïve CD4+ T cell and a probability factor corresponding to this cell type. If not blocked (all 26 non- toroidal neighborhood compartment occupied), recruited cell will be placed onto the grid. When Ag- DC and LDC are present in LN, recruitment will be up regulated by a factor (set in the parameter file).

Life span of recruited (both naïve and CM) CD4+ T cells is exponentially distributed (average specified by parameter file).

*iii. Movement*

Movement of CD4+ and CD8+ T cells are almost identical. Bound cells don’t move. If not blocked or just recruited in last move, at a probability set in parameter file (0.85 here), T cells move in preferred directions; at the probability set by the parameter file (0.10) here, T cells move to one of the non- preferred directions; otherwise it moves in opposite directions. If a T cell is blocked or just entered, T cells can move in any directions at the same probability. T cells are not allowed to move to compartments taken by FRC. Destination can be efferent lymph vessels. If destination is EL and the differentiation state allows (T cells of resting, effector, CM, and effector memory (EM) state are allowed to leave), T cells exit.

Toroidal movement: Current version of toroidal movement is: first, find the compartment on the edge of the same slice (same y value) and at the opposite position of current compartment. If it’s not a cell compartment (e.g. FRC, EF, HEV), travel circularly until we get one. For the rest of toroidal attempts of the same cell in the same movement, also travel circularly to find a cell compartment, until we reach the starting position again.

*iv. State change*

T cells have seven states: resting, bound, active, effector central memory (CM), effector memory (EM) and dead. A cell dies if time is larger than death time, (and unbind if bound).

For resting T cells: if there is a mature or licensed DC in binding radius, and cell is cognate, Naïve CD4+ T cell attempts to bind to Ag- DC or IDC at a probability (P) calculated from pMHC-II level (x), median pMHC-II (a) and a binding curve shape parameter (b).

$$P= \frac{1}{1+e^{-\frac{x-a}{b}}}$$

Set the time (16 hrs on average) and pMHC threshold (100) to unbind.

For bound T cells: CD4+ T cell start accumulating pMHC-II stimulation when they bind to a DC. If a bound T cell was CM before binding, it will accumulate stimulations faster than naïve cells (folds set in parameter file). If the bound DC dies in the same time step, T cell returns to resting state; if T cell has bound to DC for longer than unbind time or DC pMHC-II is bellow threshold, T cell unbind. If a T cell is naïve before binding, it gets activated at a probability (P) calculated from accumulated pMHC-II level (x), medium accumulated pMHC-II (c) and a priming curve shape parameter (d). Otherwise, T cell returns to resting.

$$P= \frac{1}{1+e^{-\frac{x-c}{d}}}$$

If a T cell is CM, it will always proceed to active state. If activated, life span is reset to active T cell lifetime (from an exponential distribution with mean set in the parameter file, or 96 hours for the baseline).

For active T cells: if cell has reached minimum division number (4 times), the T cell becomes effector by probability (P), which is a function of the accumulated pMHC-II stimulation (x). Parameters include medium accumulated pMHC-II required for effectors (e) and a priming curve shape parameter (f). New effector cell get reset to effector life time (exponentially distributed, with mean of 60 hrs).

$$P= \frac{1}{1+e^{-\frac{x-e}{f}}}$$

If not, and cell has lived longer than doubling time (8 hours), T cell proliferate and daughter cell is placed in Moore neighborhood. Both cells reset their lifetime.

For effector cells: cells can continue to divide up to max division number (8 for CD4+ T cells). Every time an effector T cell divides, the daughter cells can differentiate into EM cells by a probability set in the parameter file.

For CM cells: if there is a mature or licensed DC in binding radius, CM cells bind to the DC and switch to bound state.

**S1.2.2. CD8 T Cells**

*i. Constants*

Naïve ecruitment probability: 0.000408 (mm^3^)

CM ecruitment probability: 0.00204 (mm^3^)

Extra recruitment: 1.25

ProbMoveInPreferredDirection: 0.85

ProbMoveInNonOppositeDirection: 0.95

Average naïve life: 365 (day)

Binding radius: 2

Median binding pMHC-I: 150

Binding curve shape: 15

Unbinding threshold: 100

Max naïve bind time: 12 (hour)

Max naïve bind time SD: 4 (hour)

Max CM bind time: 30 (min)

Max CM bind time SD: 10 (min)

Median priming pMHC-I: 1000 (hour)

Priming curve shape: 100 (hour)

Median effector pMHC-I: 2000 (hour)

Effector curve shape: 500 (hour)

Average active life: 96 (hour)

Average effector life: 60 (hour)

Average CM life: 365 (day)

Average EM life: 60 (day)

Min division: 4

Max division: 20

Doubling time: 6 (hour)

*ii. Recruitment*

Each HEV port tries to recruit one cell at every time step. The probability that a naïve or central memory (CM) CD8+ T Cell at every time step is calculated from the product of concentration of blood naïve CD8+ T cell and a probability factor corresponding to this cell type. If not blocked (all 26 non- toroidal neighborhood compartment occupied), recruited cell will be placed onto the grid.

Life span of recruited (both naïve and CM) CD8+ T cells is exponentially distributed (average specified by parameter file).

*iii. Movement*

Movement of CD+ T cells is the same as CD4+ T cells in current setting.

*iv. State change*

Currently, rules of state change for CD8+ T cells are very similar to that of CD4+ cells. Differences are: CD8+ cells can only proceed to memory state after binding to a licensed DCs; min/max dividing count is 8/12 for CD8+ T cells.

**S1.2.3 Dendritic Cells**

*i. Constants*

Recruitment Start Time: 2 (day)

Recruitment probability: 0.06

Max DC number: 400

Average age: 5 (day)

Max time mature: 60 (hour)

Max time mature SD: 5 (hour)

Average entry age for matured: 20 (hour)

Movement interval: 4 time steps

pMHC-I half life: 25 (hour)

pMHC-II half life: 100 (hour)

Maturation pMHC extra increase: 50

Licensing probability: 0.005

Max life of licensed DC: 36 (hour)

Max life of licensed DC SD: 4 (hour)

*ii. Recruitment*

Every time to recruit DC, a random position is chosen from DC recruit ports, and T cells in that position are relocated.

State determination: pMHC value is assigned to DC at recruitment. At a probability of 0.4, newly recuited DC have pMHC of 25 ± 5, and the rest have pMHC of 250 ± 25. If pMHC is less than 50 (set in parameter file), DC is an IDC; otherwise it’s an Ag- DC. So at recruitment, Ag- DC consists about 40 percent of DCs.

If DC is immature when recruited, life span of recruited DCs are normally distributed (MaxAge ± MaxAgeSD). Current age is determined by comparing ageFactor×lifeTime and minAge. If minAge is larger, current age is set the same as minAge, otherwise, a random integer within this range is selected to be current age. So life span of DCs is uniformly distributed in (0,lifeTime- 1), which in average is 5 days.

If DC is mature when recruited, life span will be determined by (maxTimeMature ± SD) − (aveEntryAge ± SD), which in average is 40 hours.

*iii. Movement*

DC moves every 4 time steps. When bound to T cells, DCs don’t move. DC’s movement does not consider previous direction. Choose from all open destinations at the same probability regardless of the direction. When a DC moves into grid compartments occupied by T cells, these T cells are pushed away and relocated. When moving, DC can tolerate at most one FRC overlapping with it to capture the flexibility. DCs can’t leave from EL (blocked when scanning for open destinations). At boundaries, DC movement is reflective instead of toroidal.

*iv. State change*

DCs have four states: immature, mature, licensed or dead. Every time step, pMHC level decreases at a rate calculated from pMHC half life (25 hrs for pMHC-I and 100 hrs for pMHC-II).

If simulation time is larger than time for cell to die, DC unbind all T cells and die.

If cell is immature: if there is a mature or licensed DC in Moore neighborhood of 1 compartment to this DC, DC became mature at a given probability. After activation, pMHC level is set to the pMHC level of the neighboring cell + 50; life span will be determined by (maxTimeMature ± SD) (60 hours in average).

If cell is mature: if there is an effector CD4+ T cell within Moore neighborhood of 2 compartments to this Ag- DC, at certain probability (licensing probability, 0.005), DC became licensed, with pMHC increasing 150 and life span reset to (timeLicensed ± SD) (36 hours in average).

**S2. ODE blood sub- model: equations**

$$\frac{dN_{4}}{dt}=s_{N4}\left( t \right)-\delta_{N4}N_{4}+ e_{N4}^{LN} \left( 1 \right)$$

$$\frac{dE_{4}}{dt}=-\delta_{E4}E_{4}-\xi_{E4}E_{4}+e_{E4}^{LN} \left( 2 \right)$$

$$\frac{d{CM}_{4}}{dt}=-\delta_{CM4}{CM}_{4}+\alpha_{EM4}{EM}_{4}+e_{CM4}^{LN} \left( 3 \right)$$

$$\frac{d{EM}_{4}}{dt}=-\delta_{EM4}{EM}_{4}-\xi_{EM4}{EM}_{4}-\alpha_{EM4} {EM}_{4}+e_{EM4}^{LN} \left( 4 \right)$$

$$\frac{dN_{8}}{dt}=s_{N8}\left( t \right)-\delta_{N8}N_{8}+ e_{N8}^{LN} \left( 5 \right)$$

$$\frac{dE_{8}}{dt}=-\delta_{E8}E_{8}-\xi_{E8}E_{8}+e_{E8}^{LN} \left( 6 \right)$$

$$\frac{d{CM}_{8}}{dt}=-\delta_{CM8}{CM}_{8}+\alpha_{EM8}{EM}_{8}+e_{CM8}^{LN} \left( 7 \right)$$

$$\frac{d{EM}_{8}}{dt}=-\delta_{EM8}{EM}_{8}-\xi_{EM8}{EM}_{8}-\alpha_{EM8} {EM}_{8}+e_{EM8}^{LN} \left( 8 \right)$$

Equation 1 - 4 govern dynamics of naïve, effector, central memory and effector memory CD4+ T cells. Equation 5 - 8 govern dynamics of naïve, effector, central memory and effector memory CD8+ T cells. For Equation 1 and 5 respectively (t is time in days):

$$s_{N4}\left( t \right)=s_{N4,0}{\times e}^{-t\times\frac{0.05}{365}}$$

$$s_{N8}\left( t \right)= s_{N8,0}{\times e}^{-t\times\frac{0.05}{365}}$$

$e_{N4}^{LN},e_{E4}^{LN},e_{CM4}^{LN},e_{EM4}^{LN}$, $e_{N8}^{LN},e_{E8}^{LN},e_{CM8}^{LN},e_{EM8}^{LN}$ are the blood concentration changes converted from LN net output of corresponding cells. For naive and CM cells, this is the difference between the number of exited and recruited cell, per time step. For effector and EM cells, this is the number of exited cells per time step. These eight terms are not solved in the ODE system but rather are added as an initial condition before each blood time step is processed in the computational model.

# S3. Parameter values and initial conditions

This table lists all parameter values in the models. Listed first are parameters governing T cell motility and trafficking within a LN (marked with⊥); these include number and location of LN entrance and exit ports (HEVs and ELs). These values were determined in our previous study ([Gong et al., 2013](#_ENREF_10)). In this work, we added memory cells of different classes. To estimate parameters related to CD8+T cell proliferation and both CD4+ and CD8+ T cell memory differentiation (marked with ‡) we varied them to fit the model to experimental data (See Section 2.5 in the paper). The parameter set obtained from these different sources was then used as our infection baseline (“Baseline Values” column in the table below). All other parameters were fixed at their values in the previous study ([Gong et al., 2013](#_ENREF_10)).

In addition to parameter estimation, we perform sensitivity analyses to explore different model features. To this end, we chose two unique sets of parameters to be studied in intra- and inter- compartment sensitivity analyses (i.e. LN and blood related sensitivity analyses, marked with + and §, respectively). The range of the parameters varied in these analyses are shown in the “Value or Range in Sensitivity Analysis” column of Supplementary Table 3.

| Parameter (units) | | Baseline Value | Value or Range in Sensitivity Analysis | Description |
| --- | --- | --- | --- | --- |
| *General* | |  |  |  |
| ⊥ | probMovePreferredDir | 0.85 | 0.85 | Probability of moving into preferred directions |
| ⊥ | probMoveNonOppositeDir | 0.95 | 0.95 | Probability of not moving into opposite directions |
| ⊥ | probMovePreferredDirBlock | 0.6 | 0.6 | Probability of moving into preferred directions after being blocked |
| ⊥ | probMoveNonOppositeDirBlock | 0.92 | 0.92 | Probability of not moving into opposite directions after being blocked |
|  | binding radius (compartments) | 2([Miller et al., 2004a](#_ENREF_19)) | 2 | T cells within this range can bind to DCs |
| *CD4+ T cell* | |  |  |  |
|  | average life naïve (day) | 365([McCune et al., 2000](#_ENREF_18)) | 365([McCune et al., 2000](#_ENREF_18)) | Average lifetime of naïve CD4+ T cell |
| + | binding checkpoint threshold | 150([Demotz et al., 1990](#_ENREF_7)) | 100 - 200 | pMHC-II level corresponding to 50% CD4+ T cell binding probability |
|  | binding checkpoint shape | 15 | 15 | Shape of curve parameter for CD4+ T cell binding probability curve |
| +/‡ | bind time (hour) | 12 | 6 - 20 | Time CD4+ T cell bind to DCs |
|  | bind time stdev (hour) | 4 | 4 | Standard deviation of binding time |
| + | CM bind time (min) | 30 | 20 - 60 | Time CM CD4+ T cell bind to DCs |
|  | CM bind time stdev (min) | 10 | 10 | Standard deviation of CM binding time |
| +/‡ | priming checkpoint threshold (hour) | 1000 | 500 - 2000 | pMHC-II level corresponding to 50% CD4+ T cell priming probability |
| ‡ | priming checkpoint shape (hour) | 100 | 150 | Shape of curve parameter for CD4+ T cell priming probability curve |
|  | average time active (hour) | 96 | 96 | Average lifetime of active CD4+ T cells |
|  | minimum number of divisions | 4([Foulds et al., 2002](#_ENREF_9);[Miller et al., 2004b](#_ENREF_20)) | 4 | Number divisions CD4+ T cells are allowed before differentiating into effector T cells |
|  | maximum number of divisions | 8 | 8 | Maximum number of divisions allowed by CD4 T cells |
|  | double time (hour) | 6([Linderman et al., 2010](#_ENREF_16)) | 6 | Time interval between two divisions |
| +/‡ | effector checkpoint threshold (hour) | 2000 | 1500 - 3000 | pMHC-II level corresponding to 50% probability CD4+ T cell entering effector state |
| ‡ | effector checkpoint shape (hour) | 500 | 500 | Shape of curve parameter for CD4+ T cell (probability curve of entering effector state) |
|  | average time effector (hour) | 60([Sprent and Tough, 2001](#_ENREF_22)) | 60 | Average lifetime of effector CD4+ T cells |
|  | cognate frequency | 1/10000([Casrouge et al., 2000](#_ENREF_4);[Blattman et al., 2002](#_ENREF_3)) | 1/10000 | Frequency of cognate CD4+ T cells |
| + | extra recruitment | 1.25 | 1 - 1.5 | Extra recruitment during an immune response |
| +/‡ | efficiency CM | 6 | 5 - 20 | Efficiency of CM to accumulate DC signals |
| +/‡ | probability EM | 0.1 | 0.025 - 0.4 | Probability of daughter cells differentiating into EM after effector T cell division |
| *CD8+ T cell* | |  |  |  |
|  | average life naïve (day) | 365([McCune et al., 2000](#_ENREF_18)) | 365 | Average lifetime of naïve CD8+ T cell |
| + | binding checkpoint threshold | 150([Henrickson et al., 2008](#_ENREF_11)) | 100 - 200 | pMHC-I level corresponding to 50% CD8+ T cell binding probability |
|  | binding checkpoint shape | 15 | 15 | Shape parameter for CD8+ T cell binding probability curve |
| +/‡ | bind time (hour) | 12 | 6 - 20 | Time CD8+ T cell bind to DCs |
|  | bind time stdev (hour) | 4 | 4 | Standard deviation of binding time |
| + | CM bind time (min) | 30 | 20 - 60 | Time CM CD8+ T cell bind to DCs |
|  | CM bind time stdev (min) | 10 | 10 | Standard deviation of CM binding time |
| +/‡ | priming checkpoint threshold (hour) | 1000 | 500 - 2000 | pMHC-I level corresponding to 50% CD8+ T cell priming probability |
| ‡ | priming checkpoint shape (hour) | 100 | 150 | Shape parameter for CD8+ T cell priming probability curve |
|  | average time active (hour) | 96 | 96 | Average lifetime of active CD8+ T cells |
| ‡ | minimum number of divisions | 4([Foulds et al., 2002](#_ENREF_9);[Miller et al., 2004b](#_ENREF_20)) | 4 | Number divisions CD8+ T cells are allowed before advancing to effectors |
| ‡ | maximum number of divisions | 20 | 20 | Maximum number of divisions allowed |
| ‡ | double time (hour) | 6([Linderman et al., 2010](#_ENREF_16)) | 6 | Time interval between two divisions |
| +/‡ | effector checkpoint threshold (hour) | 2000 | 1500 - 3000 | pMHC-I level corresponding to 50% probability CD8+ T cell entering effector state |
| ‡ | effector checkpoint shape (hour) | 500 | 500 | Shape parameter for CD8+ T cell probability curve of differentiating into effector state |
|  | average time effector (hour) | 60([Sprent and Tough, 2001](#_ENREF_22)) | 60 | Average lifetime of effector CD8+ T cells |
|  | cognate frequency | 1/10000([Casrouge et al., 2000](#_ENREF_4);[Blattman et al., 2002](#_ENREF_3)) | 1/10000 | Frequency of cognate CD8+ T cells |
| + | extra recruitment | 1.25 | 1 - 1.5 | Extra recruitment during an immune response |
| +/‡ | efficiency CM | 6 | 5 - 20 | Efficiency of CM to accumulate DC signals |
| +/‡ | probability EM | 0.1 | 0.025 - 0.4 | Probability of daughter cells differentiating into EM after effector T cell division |
| *DC* | |  |  |  |
|  | min life immature (day) | 5([Kamath et al., 2002](#_ENREF_14)) | 5 | Average lifetime of IDC |
|  | IDC mature pMHC increment | 150 | 150 | Increment of pMHC when an IDC is activated |
|  | IDC MDC threshold | 50([Linderman et al., 2010](#_ENREF_16)) | 50 | Boundary of pMHC-II level below which a DC is IDC |
| + | pMHC-I half life (hour) | 25([Cella et al., 1997](#_ENREF_5)) | 10 - 100 | Half life of pMHC-I |
| + | pMHC-II half life (hour) | 100([Cella et al., 1997](#_ENREF_5)) | 20 - 200 | Half life of pMHC-II |
|  | unbinding threshold | 100 | 100 | pMHC-I/II level below which a CD8/CD4+ T cell unbind the DC |
|  | mature ave age entry (hour) | 20 | 20 | Average age of Ag-DC when recruited |
|  | max time mature (hour) | 60([Kamath et al., 2002](#_ENREF_14)) | 60 | Maximum age of Ag-DC |
|  | max time mature stdev (hour) | 5 | 5 | Standard deviation of max time mature |
| + | DC licensing prob. | 0.005 | 0.001 - 0.1 | Probability that an effector CD4+ T cell licenses Ag-DC |
|  | max time licensed (hour) | 36([Lanzavecchia and Sallusto, 2004](#_ENREF_15);[Lindquist et al., 2004](#_ENREF_17)) | 36 | Maximum age of LDC |
|  | max time licensed stdev (hour) | 4 | 4 | Standard deviation of LDC |
|  | recruitment prob. | 0.03 | 0.03 | Probability that an DC is recruited each time step |
| + | IDC activation prob. | 0.01 | 0.001 - 0.2 | Probability that an Ag-DC or LDC activates IDC |
|  | movement interval | 4([Miller et al., 2004b](#_ENREF_20)) | 4 | Number of time steps between each DC movement |
|  | Ag- DC recruit start (day) | 3 | 3 | Ag-DC recruitment starting time |
|  | Ag- DC recruit end (day) | 5 | 5 | Ag-DC recruitment ending time |
|  | recall response Ag- DC start (day) | 603 | 303 | Recall response Ag-DC recruitment starting time |
|  | recall response Ag- DC start (day) | 605 | 305 | Recall response Ag- DC recruitment ending time |
| ⊥ | recruit top slice | 159 | 159 | Top grid slice that DC is recruited |
| ⊥ | recruit bottom slice | 85 | 85 | Bottom grid slice that DC is recruited |
|  | size | 2 | 2 | Size of DC in each dimension |
|  | max number DC | 200 | 200 | DC recruitment pauses when DC number exceeds this point |
|  | number DC resting | 20 | 20 | Expected number of IDC at resting stage |
| *Ports* | |  |  |  |
| ⊥ | HEV count | 100 - 300 | 180 | Number of HEV port |
| ⊥ | HEV top | 154 | 154 | Top slice allowed to generate HEV ports |
| ⊥ | HEV bottom | 50 | 50 | Bottom slice allowed to generate HEV ports |
| ⊥ | EL count | 300 | 300 | Number of exit ports |
| ⊥ | EL top | 77 | 77 | Top slice allowed to generate exit ports |
| *Blood Initial Conditions* | |  |  |  |
|  | $N_{4}(0)$ (cells·mm^- 3^) | 450([Bajaria et al., 2002](#_ENREF_2)) | 450 | Initial concentration of naïve CD4+ T cells |
|  | $E_{4}(0)$ (cells·mm^- 3^) | 0 | 0 | Initial concentration of effector CD4+ T cells |
|  | ${CM}_{4}(0)$ (cells·mm^- 3^) | 0 | 0 | Initial concentration of CM CD4+ T cells |
|  | ${EM}_{4}(0)$ (cells·mm^- 3^) | 0 | 0 | Initial concentration of EM CD4+ T cells |
|  | $N_{8}(0)$ (cells·mm^- 3^) | 320([Roederer et al., 1995](#_ENREF_21)) | 320 | Initial concentration of naïve CD8+ T cells |
|  | $E_{8}(0)$ (cells·mm^- 3^) | 0 | 0 | Initial concentration of effector CD8+ T cells |
|  | ${CM}_{8}(0)$ (cells·mm^- 3^) | 0 | 0 | Initial concentration of CM CD8+ T cells |
|  | ${EM}_{8}(0)$ (cells·mm^- 3^) | 0 | 0 | Initial concentration of EM CD8+ T cells |
|  | $s_{N4}$ (cells·mm^- 3^day^- 1^) | 0.272([Bains et al., 2009](#_ENREF_1)) | 0.272 | Initial thymus output of CD4 |
|  | $s_{N8}$ (cells·mm^- 3^day^- 1^) | 0.128([Clark et al., 1999](#_ENREF_6);[Jamieson et al., 1999](#_ENREF_13)) | 0.128 | Initial thymus output of CD8 |
| *Blood* | |  |  |  |
| ⊥ | Naïve CD4+ recruit prob. (mm^3^) | 0.00058 | 0.00058 | Recruitment probability of naïve CD4 |
| ‡ | CM CD4+ recruit prob. (mm^3^) | 0.0029 | 0.0029 | Recruitment probability of CM CD4 |
| ⊥ | Naïve CD8+ recruit prob. (mm^3^) | 0.000408 | 0.000408 | Recruitment probability of naïve CD8 |
| ‡ | CM CD8+ recruit prob. (mm^3^) | 0.00204 | 0.00204 | Recruitment probability of CM CD8 |
|  | $\delta_{N4}$ (day^- 1^) | 0.0006 | 0.0006 | Naïve CD4+ death rate |
|  | $\delta_{E4}$ (day^- 1^) | 0.2 | 0.2 | Effector CD4+ death rate |
|  | $\delta_{CM4}$ (day^- 1^) | 0.0017([Homann et al., 2001](#_ENREF_12)) | 0.0017 | CM CD4+ death rate |
|  | $\delta_{EM4}$ (day^- 1^) | 0.04([Homann et al., 2001](#_ENREF_12)) | 0.04 | EM CD4+ death rate |
|  | $\delta_{N8}$ (day^- 1^) | 0.0004 | 0.0004 | Naïve CD8+ death rate |
|  | $\delta_{E8}$ (day^- 1^) | 0.2 | 0.2 | Effector CD8+ death rate |
|  | $\delta_{CM8}$ (day^- 1^) | 0.0001 | 0.0001 | CM CD8+ death rate |
|  | $\delta_{EM8}$ (day^- 1^) | 0.018([Ely et al., 2003](#_ENREF_8)) | 0.018 | EM CD8+ death rate |
| § | $\xi_{E4}$ (day^- 1^) | 0.02 | 0.1 - 0.4 | Recruitment rate of effector CD4+ to sites of infection |
| § | $\xi_{EM4}$ (day^- 1^) | 0.01 | 0.05 - 0.2 | Recruitment rate of EM CD4+ to sites of infection |
| § | $\xi_{E8}$ (day^- 1^) | 0.02 | 0.1 - 0.4 | Recruitment rate of effector CD8+ to sites of infection |
| § | $\xi_{EM8}$ (day^- 1^) | 0.01 | 0.05 - 0.2 | Recruitment rate of EM CD8+ to sites of infection |
| § | $\alpha_{EM4}$ (day^- 1^) | 0.001 | 0.001 - 0.1 | EM to CM conversion rate of CD4 |
| § | $\alpha_{EM8}$ (day^- 1^) | 0.0188([Wherry et al., 2003](#_ENREF_23)) | 0.005 - 0.1 | EM to CM conversion rate of CD8 |
|  | $e_{N4}^{LN}$ (cells·mm^- 3^timestep^- 1^) | Calculated during simulation | | Net LN naïve CD4+ output converted to change in blood concentration per time step |
|  | $e_{E4}^{LN}$ (cells·mm^- 3^timestep^- 1^) | Calculated during simulation | | Net LN effector CD4+ output converted to change in blood concentration per time step |
|  | $e_{CM4}^{LN}$ (cells·mm^- 3^timestep^- 1^) | Calculated during simulation | | Net LN CM CD4+ output converted to change in blood concentration per time step |
|  | $e_{EM4}^{LN}$ (cells·mm^- 3^timestep^- 1^) | Calculated during simulation | | Net LN EM CD4+ output converted to change in blood concentration per time step |
|  | $e_{N8}^{LN}$ (cells·mm^- 3^timestep^- 1^) | Calculated during simulation | | Net LN naïve CD8+ output converted to change in blood concentration per time step |
|  | $e_{E8}^{LN}$ (cells·mm^- 3^timestep^- 1^) | Calculated during simulation | | Net LN effector CD8+ output converted to change in blood concentration per time step |
|  | $e_{CM8}^{LN}$ (cells·mm^- 3^timestep^- 1^) | Calculated during simulation | | Net LN CM CD8+ output converted to change in blood concentration per time step |
|  | $e_{EM8}^{LN}$ (cells·mm^- 3^timestep^- 1^) | Calculated during simulation | | Net LN EM CD8+ output converted to change in blood concentration per time step |
|  | LN scaling factor | 200 | 200 | Scaling factor from model section to one LN |

+Varied in LN related sensitivity analysis; §varied in blood related sensitivity analysis

Bains, I., Antia, R., Callard, R., and Yates, A.J. (2009). Quantifying the development of the peripheral naive CD4+ T-cell pool in humans. *Blood* 113**,** 5480-5487.

Bajaria, S.H., Webb, G., Cloyd, M., and Kirschner, D. (2002). Dynamics of naive and memory CD4+ T lymphocytes in HIV-1 disease progression. *J Acquir Immune Defic Syndr* 30**,** 41-58.

Blattman, J.N., Antia, R., Sourdive, D.J., Wang, X., Kaech, S.M., Murali-Krishna, K., Altman, J.D., and Ahmed, R. (2002). Estimating the precursor frequency of naive antigen-specific CD8 T cells. *J Exp Med* 195**,** 657-664.

Casrouge, A., Beaudoing, E., Dalle, S., Pannetier, C., Kanellopoulos, J., and Kourilsky, P. (2000). Size estimate of the alpha beta TCR repertoire of naive mouse splenocytes. *J Immunol* 164**,** 5782-5787.

Cella, M., Engering, A., Pinet, V., Pieters, J., and Lanzavecchia, A. (1997). Inflammatory stimuli induce accumulation of MHC class II complexes on dendritic cells. *Nature* 388**,** 782-787.

Clark, D.R., De Boer, R.J., Wolthers, K.C., and Miedema, F. (1999). T cell dynamics in HIV-1 infection. *Advances in immunology* 73**,** 301-327.

Demotz, S., Grey, H.M., and Sette, A. (1990). The minimal number of class II MHC-antigen complexes needed for T cell activation. *Science* 249**,** 1028-1030.

Ely, K.H., Roberts, A.D., and Woodland, D.L. (2003). Cutting edge: effector memory CD8+ T cells in the lung airways retain the potential to mediate recall responses. *Journal of Immunology* 171**,** 3338-3342.

Foulds, K.E., Zenewicz, L.A., Shedlock, D.J., Jiang, J., Troy, A.E., and Shen, H. (2002). Cutting edge: CD4 and CD8 T cells are intrinsically different in their proliferative responses. *J Immunol* 168**,** 1528-1532.

Gong, C., Mattila, J.T., Miller, M., Flynn, J.L., Linderman, J.J., and Kirschner, D. (2013). Predicting lymph node output efficiency through systems biology. *Journal of Theoretical Biology*.

Henrickson, S.E., Mempel, T.R., Mazo, I.B., Liu, B., Artyomov, M.N., Zheng, H., Peixoto, A., Flynn, M.P., Senman, B., Junt, T., Wong, H.C., Chakraborty, A.K., and Von Andrian, U.H. (2008). T cell sensing of antigen dose governs interactive behavior with dendritic cells and sets a threshold for T cell activation. *Nat Immunol* 9**,** 282-291.

Homann, D., Teyton, L., and Oldstone, M.B. (2001). Differential regulation of antiviral T-cell immunity results in stable CD8+ but declining CD4+ T-cell memory. *Nature medicine* 7**,** 913-919.

Jamieson, B.D., Douek, D.C., Killian, S., Hultin, L.E., Scripture-Adams, D.D., Giorgi, J.V., Marelli, D., Koup, R.A., and Zack, J.A. (1999). Generation of functional thymocytes in the human adult. *Immunity* 10**,** 569-575.

Kamath, A.T., Henri, S., Battye, F., Tough, D.F., and Shortman, K. (2002). Developmental kinetics and lifespan of dendritic cells in mouse lymphoid organs. *Blood* 100**,** 1734-1741.

Lanzavecchia, A., and Sallusto, F. (2004). Lead and follow: the dance of the dendritic cell and T cell. *Nat Immunol* 5**,** 1201-1202.

Linderman, J.J., Riggs, T., Pande, M., Miller, M., Marino, S., and Kirschner, D.E. (2010). Characterizing the dynamics of CD4+ T cell priming within a lymph node. *J Immunol* 184**,** 2873-2885.

Lindquist, R.L., Shakhar, G., Dudziak, D., Wardemann, H., Eisenreich, T., Dustin, M.L., and Nussenzweig, M.C. (2004). Visualizing dendritic cell networks in vivo. *Nat Immunol* 5**,** 1243-1250.

Mccune, J.M., Hanley, M.B., Cesar, D., Halvorsen, R., Hoh, R., Schmidt, D., Wieder, E., Deeks, S., Siler, S., Neese, R., and Hellerstein, M. (2000). Factors influencing T-cell turnover in HIV-1-seropositive patients. *J Clin Invest* 105**,** R1-8.

Miller, M.J., Hejazi, A.S., Wei, S.H., Cahalan, M.D., and Parker, I. (2004a). T cell repertoire scanning is promoted by dynamic dendritic cell behavior and random T cell motility in the lymph node. *Proc Natl Acad Sci U S A* 101**,** 998-1003.

Miller, M.J., Safrina, O., Parker, I., and Cahalan, M.D. (2004b). Imaging the single cell dynamics of CD4+ T cell activation by dendritic cells in lymph nodes. *J Exp Med* 200**,** 847-856.

Roederer, M., Dubs, J.G., Anderson, M.T., Raju, P.A., and Herzenberg, L.A. (1995). CD8 naive T cell counts decrease progressively in HIV-infected adults. *The Journal of clinical investigation* 95**,** 2061-2066.

Sprent, J., and Tough, D.F. (2001). T cell death and memory. *Science* 293**,** 245-248.

Wherry, E.J., Teichgraber, V., Becker, T.C., Masopust, D., Kaech, S.M., Antia, R., Von Andrian, U.H., and Ahmed, R. (2003). Lineage relationship and protective immunity of memory CD8 T cell subsets. *Nature immunology* 4**,** 225-234.
